# A Horizontal Magnetic Tweezers for Studying Single DNA Molecules and DNA-Binding Proteins

**DOI:** 10.3390/molecules26164781

**Published:** 2021-08-07

**Authors:** Roberto Fabian, Santosh Gaire, Christopher Tyson, Raghabendra Adhikari, Ian Pegg, Abhijit Sarkar

**Affiliations:** 1 Department of Physics and Vitreous State Laboratory, The Catholic University of America, Washington, DC 20064, USA; gaire@cua.edu (S.G.); pegg@cua.edu (I.P.); SARKAR@cua.edu (A.S.); 2 Biomedical Engineering Department and Vitreous State Laboratory, The Catholic University of America, Washington, DC 20064, USA; 88tyson@cua.edu; 3 Department of Biology, The Catholic University of America, Washington, DC 20064, USA; adhikari@cua.edu

**Keywords:** single molecule micromanipulation techniques, horizontal magnetic tweezers, DNA-histone interactions, nucleosomes

## Abstract

We report data from single molecule studies on the interaction between single DNA molecules and core histones using custom-designed horizontal magnetic tweezers. The DNA-core histone complexes were formed using λ-DNA tethers, core histones, and NAP1 and were exposed to forces ranging from ~2 pN to ~74 pN. During the assembly events, we observed the length of the DNA decrease in approximate integer multiples of ~50 nm, suggesting the binding of the histone octamers to the DNA tether. During the mechanically induced disassembly events, we observed disruption lengths in approximate integer multiples of ~50 nm, suggesting the unbinding of one or more octamers from the DNA tether. We also observed histone octamer unbinding events at forces as low as ~2 pN. Our horizontal magnetic tweezers yielded high-resolution, low-noise data on force-mediated DNA-core histone assembly and disassembly processes.

## 1. Introduction

Histones are the basic protein unit of the nucleosome core particle. The core particle allows for the first stage of DNA compaction in eukaryotic cells, which involves the formation of a linear array of nucleosomes along the DNA [1]. The four types of histones, H2A, H2B, H3, and H4, combine to form an octameric complex comprising two copies of each type of histone. The interaction between the DNA and the histones are non-specific, with the DNA wrapped approximately 1.75 times [2] around the octameric complex. The fifth type of histone, H1 or H5, serves as a link between the adjacent nucleosomes.

Electrostatic attraction is responsible for the binding of histones to the DNA. DNA has a net negative charge on its phosphate backbone, while histones have a net positive charge in their amino terminal tails, which results in a strong electrostatic attraction. Additionally, the helix dipoles in H2B, H3, and H4 contribute to the interaction with DNA [3]. As a result, the formation of a single nucleosome results in a relatively large decrease in free energy of ~20 k_B_T [4].

The strong electrostatic interactions between DNA and histones present a great challenge to the cell’s genetic machinery. Genetic processes like DNA transcription, DNA repair, and DNA replication, which involve specialized protein machinery, require access to the sections of the DNA stored in the nucleosomes but that is rendered inaccessible by the strong binding between the DNA and the histone core particles. The application of force and torque by DNA-bound protein complexes may alter the stability of the nucleosome and may play a key role in providing access to the DNA. Interestingly, several molecular machines such as RNA polymerase and DNA polymerase have been observed to apply [5] large amounts of force in the range of ~10 pN to single DNA templates. The theoretical estimate of the minimum force, f*, needed to mechanically destabilize a nucleosome is ~2 pN [4]. This suggests that in vivo protein machines may be able to apply forces large enough to locally affect the stability of the nucleosomes.

Optical traps and magnetic tweezers have been used to investigate force-mediated nucleosome dynamics. Several studies have been performed using different procedures for histone purification and nucleosome reconstitution [6,7,8,9]. The results depend on (a) the experimental technique used, i.e., optical or magnetic tweezers, (b) the protocols used for nucleosome reconstitution, (c) the degree of control of the posttranslational modifications, (d) the absence or presence of linker histones, (e) the ionic conditions, (f) the buffer compositions, and (g) other experimental factors [6]. In Section 1.1 and Section 1.2, we summarize results from selected studies on nucleosomes using optical trapping and magnetic tweezers and briefly discuss how they have contributed to our understanding of the structure and dynamics of nucleosomes and chromatin. In Section 1.3, we introduce and motivate our study.

### 1.1. Single Molecule Studies on Nucleosomes Using Optical Traps

Optical traps and magnetic tweezers have been used to probe the structure and dynamics of chromatin. In this section, we summarize selected references on the study of single molecule experiments on nucleosomes using optical tweezers.

Cui and Bustamante [10] pioneered single molecule micromanipulation studies on nucleosomes using optical tweezers. In their experiments, they stretched and relaxed single chromatin fibers extracted from chicken erythrocytes at three different NaCl concentrations: 5 mM, 40 mM, and 150 mM. Their results established that the force versus extension curves of single chromatin fibers are reversible at forces less than 6 pN and that are irreversible at forces greater than 20 pN in all three NaCl concentrations. Nucleosome disassembly events were not reported in their experiments.

Following this study, Bennink et al. [11] performed optical trapping experiments on single chromatin fibers reconstituted from λ-DNA and Xenopus laevis egg extract. They were able to detect nucleosome disruption events starting at 20 pN and up to 40 pN. These were associated with three disruption lengths 65 nm, 130 nm, and 195 nm, which were interpreted as the signatures of one, two, and three nucleosome disassembly events, respectively.

Brower-Toland et al. [12] investigated single nucleosomes using optical tweezers with nucleosomes reconstituted from single DNA molecules and core histones (without linker histones) purified using the salt dialysis method. The DNA tethers included 17 tandem repeats of 5S positioning sequences. They found a ~25 nm disruption length of DNA per nucleosome at forces less than 15 pN and ~27 nm disruption lengths for higher forces.

Using optical tweezers, Gemmen et al. [13] investigated nucleosome arrays formed from DNA tethers with random sequences, core histones (without linker histones), the histones chaperone NAP1, and the remodeling factor ACF. They reported that the length released per nucleosome unbinding event changed from ~18 nm to ~31 nm from 5 pN to 65 pN. They also found the average force needed to unravel a single nucleosome was dependent on the NaCl concentration of the buffer, which they suggested reflected variations in the binding affinity of the histone core particle to different sequences of the DNA.

Claudet et al. [14] used optical tweezers to study nucleosome arrays reconstituted using salt dialysis. A DNA template with 12 tandem repeats of the 5S nucleosome positioning sequence and core histones (without linker histones) was used. They also conducted complementary studies using nucleosome arrays extracted from chicken erythrocytes. A total of two DNA disruption lengths of 25 nm and 50 nm were reported in their studies. Disruption events were observed to occur at forces ≥ 20 pN in both types of nucleosome arrays.

Pope et al. [15] used an optical tweezers technique to stretch single chromatin fibers assembled from λ-DNA and Xenopus laevis egg extract at different loading rates. They reported three disruption lengths: 30 nm, 59 nm, and 117 nm. All three disruption lengths occurred at forces ≥ 20 pN.

Mihardja et al. [16] investigated single mononucleosomal fibers using optical tweezers. The mononucleosomal DNA was assembled from 2582 bp DNA containing a single 601 positioning sequences and core histones extracted from chicken erythrocytes. In their experiments, they observed two disruption lengths: 21 nm at forces starting at 3 pN and 22 nm at forces between 8 and 9 pN. They interpreted these as two stages of nucleosome disassembly. The first stage represented the release of the outer wrap of the nucleosomal DNA while the second stage represented the unwrapping of the remaining inner wrap of the nucleosomal DNA.

Bussiek et al. [17] studied single chromatin fibers reconstituted from two types of DNA templates, core histones (no linker histones), and the NAP1/ACF system. The two DNA templates were heterogenous DNA, i.e., DNA with a random sequence, and tandem repeat alpha satellite DNA. They extended the individual fibers using optical tweezers. They found that the disruption length in both DNA templates were almost same, ~23 nm for heterogenous DNA and ~24 nm for alpha satellite DNA. However, the disruption force in the alpha satellite DNA was 26 pN, higher than the heterogenous DNA, which showed a disruption force of 22 pN.

### 1.2. Single Molecule Studies on Nucleosomes Using Magnetic Tweezers

Leuba et al. [18] were the first to study nucleosome arrays using vertical magnetic tweezers. In their experiments, they used chromatin fibers reconstituted from λ-DNA, core histones (without linker histones), and the chaperone protein NAP1. The core histones were purified from chicken erythrocytes. The role of the chaperone protein was to allow proper nucleosome formation during the interaction between the core histones and the DNA template. Interestingly, they did not observe tether compaction (due to binding of histone octamers) at forces higher than 10 pN.

Yan et al. [19] conducted experiments on single nucleosome arrays reconstituted from λ-DNA and Xenopus laevis egg extracts using a combination of vertical magnetic tweezers and horizontal magnetic tweezers. They performed the experiments in ATP-depleted extracts and in an extract with ATP. When ATP was present, they observed full nucleosome disassembly at 2 pN. In the experiments where ATP was absent, they observed nucleosome disassembly at 3.5 pN with a disruption length of 50 nm.

Using vertical magnetic tweezers, Simon et al. [20] investigated single nucleosome arrays with posttranslational modifications (PTM) in the core histones. They used nucleosome arrays containing H3 (K5ac), H3 (K115ac, K122ac), and H4 (K77ac, K79ac). They also used nucleosome arrays without PTMs on the core histones. All of the four nucleosome arrays were assembled on ~3 kbp DNA molecules with 17 high-affinity nucleosomes positioning sequences (NPS). Nucleosomes were constituted from DNA and core histones (without linker histones) using the salt dialysis method. The PTM histones were synthesized in their laboratory. Nucleosome disassembly events were observed at forces up to 29 pN with disruption lengths of ~24 nm to ~26 nm for the presence of posttranslational modification and ~24 nm in the absence of posttranslational modifications.

Vlijm et al. [21] studied individual nucleosome arrays using vertical magnetic tweezers. The arrays were reconstituted by incubating 8 kbp DNA, core histones (without linker histones), and NAP1. The core histones were purified from Drosophila. They found a DNA compaction length of 56 nm per nucleosome assembly at a force of ~0.3 pN, while nucleosome disassembly was observed at forces from 3 pN to 17.5 pN with a disruption length of ~25 nm.

Kaczmarczyk et al. [22] studied nucleosome–nucleosome interactions using vertical magnetic tweezers. They used two types of DNA tethers with 4535 bp and 4985 bp, respectively. Each DNA molecule contained 15 tandem repeats of the Widom 601 sequence and core histones with mutant versions of histones H2A (H2A-E64C) and H4 (H4-V21C) from Xenopus laevis egg extract. Nucleosome arrays were reconstituted using either the standard salt dialysis method or the salt dialysis method modified to induce the formation of nucleosome–nucleosome disulfide bonds along the nucleosome arrays. They found that the force needed to destabilize nucleosome–nucleosome interactions occurred from 3 pN to 4 pN in both types of chromatin fibers. At these forces, the fibers were believed to have undergone an unstacking transition. Further analysis revealed that the unstacking transition in the 4985 bp fibers was more gradual than in the 4535 bp fibers. They attributed these results to the unfolding of the higher order chromatin fiber structure. The 4535 bp fibers had a two-start zig-zag supercoiled structure, while the 4985 bp fibers had a one-start solenoidal structure. They also observed nucleosome disassembly at forces between 7 pN to 20 pN with a disruption length of ~25 nm in both classes of chromatin fibers.

Ordu et al. [23] investigated the dynamics of the handedness flipping of single tetrasome fibers using Freely Orbiting Magnetic Tweezers (FOMT). The handedness flipping of tetrasomes refers to the spontaneous twisting of the DNA wrapped around in the tetrasomes from left-handed to right-handed, and vice versa. They used tetrasome fibers reconstituted from 1.97 kbp DNA and histones H3 and H4 (untreated tetrasomes) and also used fibers assembled from 1.97 kbp DNA and the histone variants of H3, H3.1, and H3.3 (treated tetrasomes). In both cases, NAP1 was used, and the treated and untreated tetrasomes were reconstituted using the salt dialysis method. Their results showed that the treated tetrasomes exhibited the same structural properties as the untreated tetrasomes. However, the flipping kinetics of the treated tetrasomes were three times slower compared to the untreated tetrasomes, suggesting that treated histones could stably maintain their handedness longer than untreated tetrasomes.

### 1.3. Nucleosome Stability Explored Using Horizontal Magnetic Tweezers

The results of these selected studies show wide variation in the data even when the experiments were performed using the same experimental technique. In our experiments, we reconstituted DNA-histone octamer complexes from λ-DNA, core histones, and NAP1, resulting in nucleosomes located at random positions on the DNA tether. We used histones H2A, H2B, H3, and H4; H1 and H5 were not used in our studies. These arrays were mechanically micromanipulated using the horizontal magnetic tweezers apparatus described in [24]. Briefly, linear DNA was tethered to superparamagnetic beads on both ends with one of the beads also connected to a stiff biotinylated micropipette while the other bead was suspended in buffer. The distance between a fixed permanent magnet and the suspended bead could be adjusted, which allowed us to control the force applied to the tether. The design of our magnetic tweezers took advantage of the horizontal geometry of the tethered DNA, which allowed differential detection on the centroids of the beads. The sample cell design allowed buffer exchange and the introduction of DNA-binding proteins on demand. The goal of our study was to analyze the stability of our in situ-assembled DNA-core particle arrays to mechanical perturbations and to compare our results to studies reported in the literature (and partially reviewed above.)

## 2. Results and Discussion

The basic principle of our single molecule experiments was to (1) allow the binding of histone octamers onto a naked DNA tether held under tension less than 4 pN and then (2) to raise the force to destabilize the DNA-histone complexes and map the step-wise decompaction events. These experiments were conducted until the full DNA length was recovered. The binding of the core histones to the DNA tether was observed via the visible compaction of the DNA tether. The experimental procedure is described in more detail in the Methods section. Additionally, we have provided a schematic of the horizontal magnetic tweezers—see Materials and Methods section.

**Figure 1:**
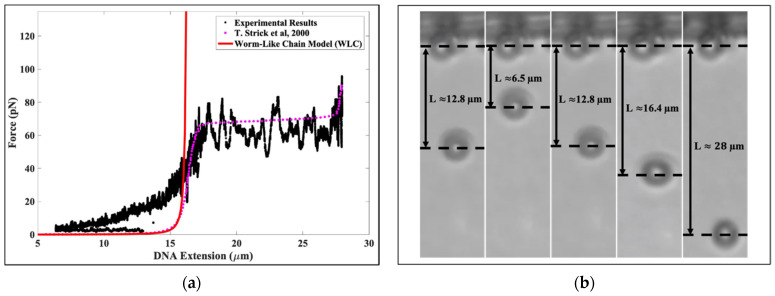
(**a**) The force versus extension for a DNA-histone complex under tension. The solid black circles represent our experimental results. The solid pink circles represent the experimental force–extension data for λ-DNA obtained using optical tweezers—see Ref. [26]. The solid red line represents the force–extension behavior of a worm-like chain model under tension. (**b**) A total of five snapshots from an experiment showing the compaction and force-induced decompaction of histones bound to a single DNA tether. From left to right, the first snapshot shows naked DNA (DNA before introduction of histones) with an end-to-end extension of ≈12.8 μm corresponding to a force of ≈2.2 pN. The second snapshot shows that the extension of the λ-DNA has reduced to ≈6.5 μm after binding by histones at a force of ≈2.5 pN. The third snapshot shows that the tether has re-extended to ≈12.8 μm at a force of ≈16.4 pN. The fourth snapshot shows that λ-DNA has reached its contour length of ≈16.4 μm (here, the force is ≈40 pN.) The fifth snapshot shows the λ-DNA beyond the overstretching transition, where it has stretched to ≈28 μm; this corresponds to a force of ≈74 pN.

Figure 1a presents a typical force vs. extension plot of a histone-core particle-studded DNA tether under tension. Additionally, plotted for calibration purposes are (1) the modified worm-like chain force–extension relationship for double stranded DNA (dsDNA) (red line) [25] and (2) single molecule DNA pulling data from Strick et al. [26], represented by solid pink circles. The solid black circles represent our experimental data on the micromechanical response of the compacted DNA to the applied tension. From the graph, at forces greater than 40 pN, when all bound octamers have been mechanically removed, our experimental data are consistent with the experimental results of Strick et al. [26], indicating that we started with a single DNA tether. Figure 1b shows five snapshots from an experiment with compacted tethers. From left to right, the first snapshot shows that the length of the DNA was ~12.8 μm at a force ~2.2 pN before the core histones and NAP1 were introduced. The second snapshot shows that the length of the DNA decreased to ~6.5 μm at a force of ~2.5 pN after the core histones and NAP1 were introduced. The third snapshot shows that the length of the DNA increased to ~12.8 μm with corresponding force of ~16.4 pN. The fourth snapshot shows that the length of the DNA was ~16.4 μm, which is the contour length of λ-DNA, with a corresponding force of ~40 pN. Finally, the fifth snapshot shows the DNA overstretching transition, in which the length of the DNA increased to ~28 μm, which is ~70% longer than the contour length of λ-DNA. This last observation also serves as an additional check that the tether was indeed a single λ-DNA molecule.

**Figure 2:**
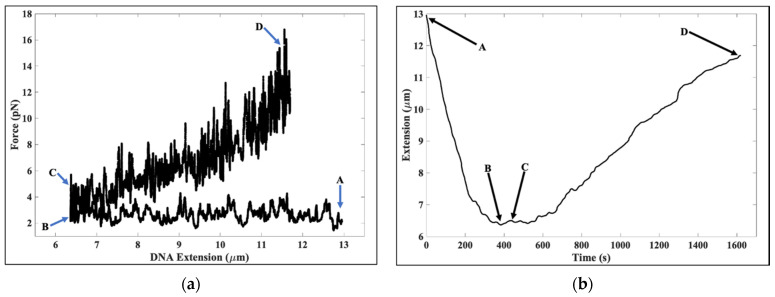
(**a**) The arrows from A to B show the region where upon binding to histones, the length of the λ-DNA decreases from ~12.8 μm to ~6.4 μm at forces ranging from ≈2.2 pN at A to ≈2.7 pN at B. The arrows from B to C indicate the region where the forces were slowly increased from ~2.7 pN to ~3.3 pN, with the corresponding change in DNA extension from ~6.4 μm to ~6.5 μm. The arrows from C to D demarcate the region where the DNA tether was subject to forces from ≈3.3 pN to ≈12.5 pN, with the corresponding DNA extension increasing from ~6.5 μm to ~11.7 μm. (**b**) Extension vs. time for the data in part (**a**). Arrow A corresponds to a DNA extension of ~12.8 μm, arrow B corresponds to a DNA extension of ~6.4 μm, arrow C corresponds to a DNA extension of ~6.5 μm, and arrow D corresponds to a DNA extension of ~11.7 μm. In regions A to B, we moved the magnet away from the λ-DNA at speeds between 0.5 μm/s and 1 μm/s. In regions B to D, we moved the magnet towards the tethered DNA at a speed of 0.5 μm/s.

Figure 2a shows the experimental data presented in Figure 1a in more detail. In Figure 2a, the arrows from A to B indicate the start and end lengths as the tether underwent compaction due to the binding of the core histones from ~12.8 μm to ~6.4 μm (The force was slightly increased from ~2.2 pN to ~2.7 pN near the end of the compaction to prevent the binding of the suspended bead to the biotinylated glass micropipette.) In the region demarcated by arrows B to C, the force was slowly increased from ~2.7 pN to ~3.3 pN, with a slight increase in the tether length from ~6.4 μm to ~6.5 μm. The compacted DNA began to re-extend after arrow C. Arrows C to D define the region where the DNA tether was subject to forces between ~3.3 pN to ~12.5 pN and where DNA tether extension increased from ~6.5 μm to ~11.7 μm.

Figure 2b is the corresponding extension versus time plot of Figure 2a. In Figure 2b, the length of the tethered DNA at arrow A is ~12.8 μm, the length of the tethered DNA at arrow B is ~6.4 μm, the length of the tethered DNA at arrow C is ~6.5 μm, and the length of the tethered DNA at arrow D is ~11.7 μm. In regions A to B, we moved the magnet away from the DNA tether at speeds between 0.5 μm/s and 1 μm/s. In regions B to D, we moved the magnet towards the DNA tether at a speed of 0.5 μm/s.

**Figure 3:**
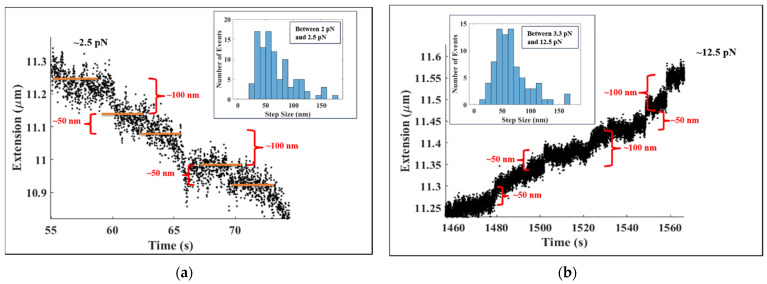
(**a**) The figure shows tether extension changes corresponding to histone-DNA complexation events at a force of ~2.5 pN. These data are from the region between Arrow A and Arrow B of Figure 2a presented at a higher resolution. Note the decrease of the length of the λ-DNA in multiple integrals of ~50 nm, suggesting the binding of core histone octameric units to the DNA tether. The horizontal lines (orange) indicate the step plateaus. Inset: Distribution of step sizes during the compaction of the DNA tether from 1 s to 400 s where the force range was between 2 pN and 2.5 pN. There were 99 steps in the distribution. Among them, 52 steps—more than 50% of the total—fall between 30 nm and 60 nm, with mean step size of ~47.2 ± 9.7 nm. Additionally, between 85 nm and 115 nm, we found 18 steps (~18% of the total) with a mean step size of ~99.9 ± 9.1 nm. (**b**) The figure shows the disruption events at a force of ~12.5 pN—data from Arrow D of Figure 2b—at higher resolution. Observe the extension increases upon each disruption step occurring in multiple integrals of ~50 nm, suggesting the mechanically induced unraveling of nucleosomes from the DNA tether. Inset: Distribution of step sizes during the decompaction of the DNA tether from 533 s to 1616 s, where the force range was between 3.3 pN and 12.5 pN. There were 80 steps in the distribution. Among them, 41 steps—more than 50% of the total—fell between 40 nm and 70 nm, with mean step size of ~53.7 ± 9.7 nm. Additionally, between 85 nm and 115 nm, we found 12 steps (~14% of the total) with a mean step size of ~97.5 ± 8.6 nm.

Figure 3a shows the binding events that produced the initial tether compaction between arrows A and B in Figure 2a at higher resolution. These binding events were at a (nearly) constant force of ~2.5 pN. In Figure 3a, the length of the DNA tether decreases in multiple integrals of ~50 nm at a force of ~2.5 pN. Note that a decrease of ~50 nm is what would be expected from the binding and wrapping of DNA by a single histone octameric unit; similarly, a decrease of ~100 nm is consistent with the simultaneous binding and subsequent winding of DNA by two histone octamers. The inset shows the step size histogram for forces between 2 pN and 2.5 pN with corresponding times from 1 s to 400 s. The histogram has a total of 99 steps. Considering only the steps between 30 nm and 60 nm, we found the mean step size to be 47.2 ± 9.7 nm. For the steps between 85 nm and 115 nm, the mean step size was ~99.9 ± 9.1 nm. Of the 99 steps, ~68% of the steps (~50% between 30–60 nm and ~18% between 85–115 nm) were contained in these two bin intervals. Figure 3b zooms in on the unbinding events in Figure 2b at arrow D, where the force is ~12.5 pN. In Figure 3b, the length of the DNA increases in multiples of ~50 nm, which is the expected signature of the mechanically induced removal of one (~50 nm extension increase), two (~100 nm extension increment), or more octamers bound to the λ-DNA tether. The inset shows the step size histogram for forces between 3.3 pN and 12.5 pN with the corresponding times from 533 s to 1616 s. The histogram has a total of 80 steps. The steps between 40 nm and 70 nm had a mean step size of 53.7 ± 9.7 nm, while the mean step size for the steps between 85 nm and 115 nm was ~97.5 ± 8.6 nm. Of the 80 steps, ~64% of the steps (~50% between 40–70 nm and ~14% between 85–115 nm) were contained in these two bin intervals.

**Figure 4:**
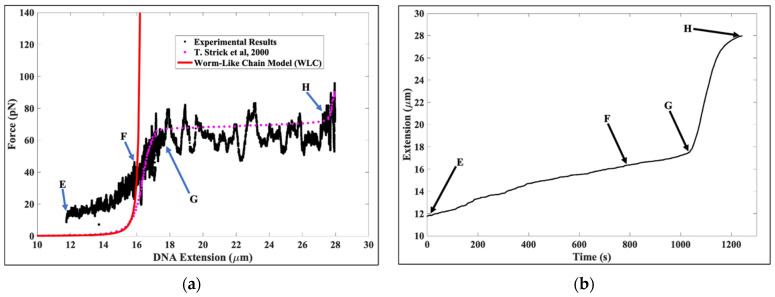
(**a**) This figure presents the force–extension response of a single DNA tether complexed to histones in the decompaction phase of the experiment. These data are a continuation of the data presented in Figure 2a,b. Between Arrows E to F, the force was gradually increased from ~12.5 pN to ~40 pN, with tether extension increasing from ~11.7 μm to ~16.4 μm. In the region bounded by Arrows F and G, the DNA tether was subjected to forces from ~40 pN to ~63 pN, with corresponding DNA extension increasing from ~16.4 μm to ~17.5 μm. The region bordered by Arrows G and H shows the DNA undergoing an overstretching transition with start and end forces of ~63 pN to ~74 pN, respectively (extension increasing from ~17.5 μm to ~28 μm, respectively). (**b**) This figure shows the DNA extension versus time from Figure 4a. The DNA extension is ~11.7 μm for Arrow E, ~16.4 μm for Arrow F, ~17.5 μm for Arrow G, and ~28 μm for Arrow H. The speed at which the magnet was moved away from the tether between Arrows E and H was ~0.5 μm/s.

Figure 4a recapitulates the data in Figure 1a. The region beyond E corresponds to forces greater than 12.5 pN. The arrows from E to F demarcate a region of gradually increasing force from ~12.5 pN to ~40 pN, with the corresponding DNA extension increasing from ~11.7 μm to ~16.4 μm. This is the region where the DNA tether approached its full contour length. Between Arrows F and G, the DNA tether was subjected to forces from ~40 pN to ~63 pN, which stretched the DNA in a Hookean manner from its zero-force contour length of ~16.4 μm to ~17.5 μm. From Arrows G to H, the DNA tether underwent the overstretching transition with forces from ~62 pN to ~74 pN. The initial DNA extension of ~17.5 μm increased at the end of the transition zone to ~28 μm, approximately 170% of the original zero-force contour length, as expected from the well-characterized DNA overstretching transition. In Figure 4a, we also observe that the region between Arrows F to H show that our experimental data are broadly consistent with the results of the optical tweezers single molecule experiments of Strick et al. [26], which serves as a further calibration check.

Figure 4b is the extension vs. time profile corresponding to Figure 4a. In Figure 4b, the DNA extensions at the arrows are ~11.7 μm at E; ~16.4 μm at F; ~17.5 μm at G; and ~28 μm at H. From arrows E to H, we moved the magnet towards the DNA tether at a speed of 0.5 μm/s.

**Figure 5:**
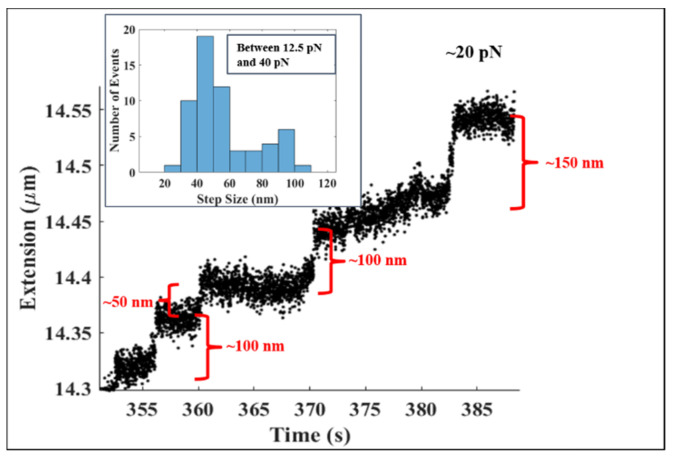
This figure shows the rupture of core histones from λ-DNA at a force of ~20 pN. The simultaneous release of core histones occurred in quantized step sizes of integral multiples of ~50 nm. Inset: Distribution of step sizes during the decompaction of the DNA tether from 10 s to 500 s, where the force ranged between 12.5 pN and 40 pN. There were 59 steps in total, with the distribution showing a bimodal structure. Over 60% of the total steps were found to have step sizes between 30 nm and 60 nm with a mean step size of ~45.1 ± 8.6 nm. Additionally, we found 11 steps (~18% of the total) between 85 nm and 115 nm with a mean step size of ~90.9 ± 6.9 nm.

Figure 5 shows the extensional response of DNA-core histone complexes around ~20 pN. A rapid extension jump was observed in integer multiples of ~50 nm. Note that the jumps were more distinct at higher forces compared to the steps at the lower forces shown in Figure 3a,b. The inset shows the step size histogram for the forces between 12.5 pN and 40 pN with the corresponding times from 10 s to 500 s. The histogram has a total of 59 steps. For the steps between 30 nm and 60 nm, we found the mean step size to be 45.1 ± 8.6 nm, while between 85 nm and 115 nm, the mean step size was ~90.9 ± 6.9 nm. This histogram has 59 steps in total, out of which ~60% were found to be between 30–60 nm, while ~18% had step sizes in the range 85–115 nm. These two bin intervals encompassed ~78% of all the steps in the distribution.

Magnetic tweezers were used to study nucleosome fibers under tension. The most widely used magnetic tweezers are the vertical magnetic tweezers. Based on our survey of the literature, we are not aware of another research group that has published micromanipulation data on reconstituted DNA-histone complexes using transverse magnetic tweezers. In our experiments the octamer-studded tethers assembled from λ-DNA, core histones (no linker histones), and NAP1 showed assembly events with extension decreases of ~50 nm at a force ~2.5 pN and disassembly events with disassembly steps of ~50 nm (and integer multiples) at forces from ~3.3 pN to ~40 pN. The use of our horizontal magnetic tweezers can be extended to study the interaction between single DNA molecules and non-native histones.

## 3. Materials and Methods

**Figure 6:**
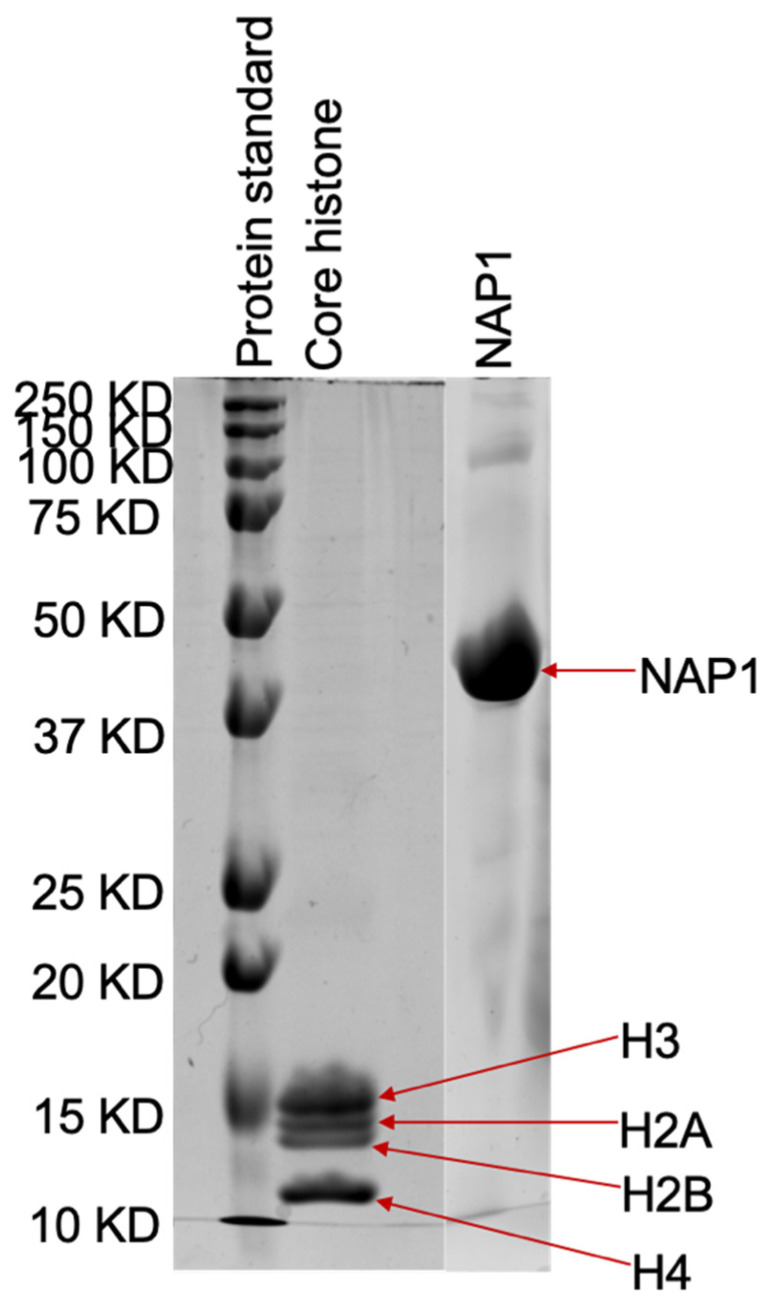
SDS-PAGE analysis of core histones and NAP1. Approximately 7.2 μg of core histones and 40 μg of NAP1 were loaded on of 15% gel and were stained with coomassie-blue after electrophoresis. The numbers on the left side indicate the molecular weight of marker proteins.

The core histones (SRP6590, Millipore Sigma, St. Louis, MO, USA) and NAP1 (14-837, Millipore Sigma) were purchased from Millipore Sigma. The coomassie-blue stained gel from the SDS-PAGE of the core histones and NAP1 showed that their molecular weights were consistent with prior expectations (Figure 6). Core histone solutions were aliquoted into small tubes at 4 °C. The aliquoted volume of the core histones was adjusted in such a way that adding 200 μL of 1× TE, 150 mM NaCl buffer produced a concentration of 0.1 mg/mL of core histones in the solution. The aliquoted solution was stored at −80 °C. NAP1 solution was aliquoted into small tubes and kept in ice. The aliquoted volume of the NAP1 was adjusted in such a way that by adding 200 μL of 1× TE, the 150 mM NaCl buffer produced a concentration of 0.2 mg/mL of NAP1. The aliquoted solution was stored at −80 °C.

**Figure 7:**
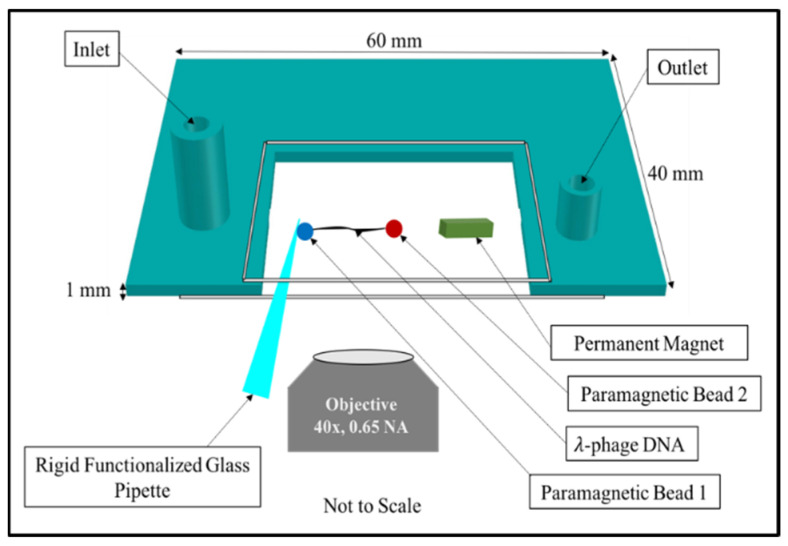
This figure shows a schematic diagram for the horizontal magnetic tweezers. Each end of the λ-DNA was attached to a superparamagnetic bead coated with streptavidin. One bead was also attached to the surface of the functionalized glass pipette and the other bead was suspended near a 3 mm × 2 mm × 1 mm neodymium bar magnet attached to the floor of the sample cell. The inlet and outlet of the sample cell were used to introduce core histones and NAP 1 proteins and for buffer exchange. The sample cell was composed of a 3D custom printed spacer and two coverslips, one with the dimensions of 22 mm × 40 mm (glued at the bottom of the spacer forming the floor of the sample cell) and another with the dimensions of 22 mm × 22 mm (glued to the top of the spacer, forming the ceiling of the sample cell).

All micromanipulation experiments were performed using the horizontal magnetic tweezers described in Ref. [24]. Figure 7 shows the schematic diagram of the horizontal magnetic tweezers. Briefly, we first functionalized the 12 base polynucleotide overhangs of the λ-DNA tether. Next, each of the two overhangs were ligated to an oligomer that contained five biotins. Third, we prepared the sample cell. The sample cell was constructed using two coverslips, a custom 3D-printed spacer, and a bar magnet. The two coverslips were glued to the top and bottom of the spacer, forming the ceiling and the floor of the sample cell. The sides of the spacer formed three of the four sides of the sample cell; one side was left open to allow for the insertion of the pipette. The spacer had an inlet and outlet for buffer exchange. The bar magnet was glued to the floor of the sample cell. Fourth, we incubated the 5 μL end-functionalized DNA in 1× Tris-EDTA (TE) and 150 mM NaCl buffer with prewashed 2.8 μm diameter superparamagnetic beads (11205D, Thermo Fisher Scientific) coated with streptavidin. After incubation, 5 μL of the DNA-bead construct was introduced through the sample cell loaded with 1× Tris-EDTA (TE) and 150 mM NaCl buffer. Fifth, we integrated the DNA-loaded sample cell and surface-functionalized glass pipette as described in [24] into the horizontal magnetic tweezers. The horizontal magnetic tweezers were composed of an optical microscope and a motorized micromanipulator. The optical microscope was attached to a camera, and the camera was connected to a computer for instrument control, data acquisition, and processing. Sixth, we focused the pipette tip and determined the distance between the pipette tip and the bar magnet. (See Ref. [24] for details). At this point, we began the search for DNA-tethered beads. Once a DNA-tethered bead was found, we moved the magnet closer to the suspended bead at 10 μm/s until it underwent the DNA overstretching transition. This confirmed that the tether consisted of a single DNA molecule. We then brought the tethered bead to a position between 1300 μm and 1500 μm from the magnet where the force was less than 4 pN.

In parallel, we prepared the core histones and NAP1 solutions as follows: We first thawed the previously aliquoted tubes of core histones and NAP1 solution stored at −80 °C and stored them in ice. The core histones and NAP1 solutions were then mixed together in 200 μL of 1× TE and 150 mM NaCl buffer. This solution contained core histones with a concentration of 0.1 mg/mL and NAP1 with a concentration of 0.2 mg/mL; henceforth, we will refer to this as the “protein solution”. The protein solution was incubated for 2 min at room temperature and was then loaded into Tygon tubing and placed in the inlet of the sample cell.

The two syringe pumps were connected to the inlet and outlet of the sample cell, respectively, and were then activated. The inlet rate was set at 5 μL/min, while the outlet rate was fixed to 12 μL/min. We recorded videos of the experiments at a frame rate of 120 Hz.

Once the beginning of the compaction process was visually observed, we turned off both syringe pumps and then moved the magnet away from the DNA tethered bead pair at a speed of 1 μm/s to a distance of 1500–1600 μm from the suspended bead, where the force was less than 3 pN. This allowed the histones to bind to the DNA at a force lower than 4 pN. We incrementally increased the force by moving the magnet towards the compacted tether at a rate of 0.5 μm/s. This was continued until the DNA tether either broke or detached from the glass surface.

Custom image processing and particle tracking algorithms were run offline to determine the force and extension of the DNA-histone complexes and to detect step-like features in the data.

## Data Availability

The data presented in this study are available on request from the corresponding author.

## Sample Availability

Samples of the compounds are not available from the authors.

## References

[1] Kornberg R.D., Thomas J.O. (1974). Chromatin structure; oligomers of histones. Science.

[2] Luger K., Mader A., Richmond R., Sargent D., Richmond T. (1977). Crystal structure of the nucleosome core particle at 2.8 a resolution. Nature.

[3] Widom J. (1977). Chromatin: The nucleosome unwrapped. Curr. Biol..

[4] Marko J.F., Siggia E.D. (1997). Driving proteins off DNA using applied tension. Biophys. J..

[5] Bustamante C., Cheng W., Mejia Y.X. (2011). Revisiting the central dogma one molecule at a time. Cell.

[6] Bednar J., Dimitrov S. (2011). Chromatin under mechanical stress: From single 30 nm fibers to single nucleosomes. FEBS J..

[7] Mack A.H., Schlingman D.J., Ilagan R.P., Regan L., Mochrie S.G. (2012). Kinetics and thermodynamics of phenotype: Unwinding and rewinding the nucleosome. J. Mol. Biol..

[8] Lavelle C., Victor J.M., Zlatanova J. (2010). Chromatin fiber dynamics under tension and torsion. Int. J. Mol. Sci..

[9] Ngo T.T., Zhang R., Zhou R., Yodh J.G. (2015). Asymmetric unwrapping of nucleosome under tension directed by DNA local flexibility. Cell.

[10] Cui Y., Bustamante C. (2000). Pulling a single chromatin fiber reveals the forces that maintain its higher-order structure. Proc. Natl. Acad. Sci. USA.

[11] Bennink M.L., Leuba S.H., Leno G.H., Zlatanova J., de Grooth B.G., Greve J. (2001). Unfolding individual nucleosomes by stretching single chromatin fibers with optical tweezers. Nat. Struct. Biol..

[12] Brower-Toland B.D., Smith C.L., Yeh R.C., Lis J.T., Peterson C.L., Wang M.D. (2002). Mechanical disruption of individual nucleosomes reveals a reversible multistage release of DNA. Proc. Natl. Acad. Sci. USA.

[13] Gemmen G.L., Sim R., Haushalter K.A., Ke P.C., Kadonaga J.T., Smith D.E. (2005). Forced unravelling of nucleosomes assembled on heterogenous DNA using core histones, NAP-1, and ACF. J. Mol. Biol..

[14] Claudet C., Angelov D., Bouvet P., Dimitrov S., Bednar J. (2005). Histone octamer instability under single molecule experiment conditions. J. Biol. Chem..

[15] Pope L.H., Bennink M.L., van Leijenhorst-Groener K.A., Nikova D., Greve J., Marko J.F. (2005). Single chromatin fiber stretching reveals physically distinct populations of disassembly events. Biophys. J..

[16] Mihardja S., Spakowitz A.J., Zhang Y., Bustamante C. (2006). Effect of force on mononucleosomal dynamics. Proc. Natl. Acad. Sci. USA.

[17] Bussiek M., Hoischen C., Diekmann S., Bennink M.L. (2009). Sequence-specific physical properties of African green monkey alpha-satellite DNA contribute to centromeric heterochromatin formation. J. Struct. Biol..

[18] Leuba S.H., Karymov M.A., Tomschik M., Ramjit R., Smith P., Zlatanova J. (2003). Assembly of single chromatin fibers depend on the tension in the DNA molecule: Magnetic tweezers study. Proc. Natl. Acad. Sci. USA.

[19] Yan J., Maresca T.J., Skoko D., Adams C.D., Xiao B., Christensen M.O., Heald R., Marko J.F. (2007). Micromanipulation studies of chromatin fibers in Xenopus egg extracts reveal ATP-dependent chromatin assembly dynamics. Mol. Biol. Cell.

[20] Simon M., North J.A., Shimko J.C., Forties R.A., Ferdinand M.B., Manohar M., Zhang M., Fishel R., Ottesen J.J., Poirier M.G. (2011). Histone fold modifications control nucleosome unwrapping and disassembly. Proc. Natl. Acad. Sci. USA.

[21] Vlijim R., Smithshuijzen J.S.J., Lusser A., Dekker C. (2012). NAP-1 assisted nucleosome assembly on DNA measured in real time by single molecule magnetic tweezers. PLoS ONE.

[22] Kaczmarczyk A., Allahverdi A., Brouwer T.B., Nordenskiold L., Dekker N.H., van Noort J. (2017). Single-molecule force spectroscopy on histone H4 tail-cross-linked chromatin reveals fiber folding. J. Biol. Chem..

[23] Ordu O., Kremser L., Lusser A., Dekker N.H. (2018). Modification of the histone tetramer at the H3-H3 interface impacts tetrasome conformations and dynamics. J. Chem. Phys..

[24] Fabian R., Tyson C., Tuma P.L., Pegg I.L., Sarkar A. (2018). A horizontal magnetic tweezers and its use for studying single DNA molecules. Micromachines.

[25] Marko J.F., Siggia E.D. (1995). Stretching DNA. Macromolecules.

[26] Strick T., Allemand J., Croquette V., Bensimon D. (2000). Twisting and stretching single DNA molecules. Prog. Biophys. Mol. Biol..

